# Adjuvant chemotherapy after surgery for pancreatic ductal adenocarcinoma: retrospective real-life data

**DOI:** 10.1186/s12957-019-1732-3

**Published:** 2019-11-09

**Authors:** Sophia Chikhladze, Ann-Kathrin Lederer, Lampros Kousoulas, Marilena Reinmuth, Olivia Sick, Stefan Fichtner-Feigl, Uwe A. Wittel

**Affiliations:** 1 Department of General and Visceral Surgery, Medical Center - University of Freiburg, Faculty of Medicine, University of Freiburg, Hugstetter Straße 55, 79106 Freiburg im Breisgau, Germany; 2 Center for Complementary Medicine, Institute for Infection Prevention and Hospital Epidemiology, Medical Center - University of Freiburg, Faculty of Medicine, University of Freiburg, Breisacher Straße 115b, 79106 Freiburg im Breisgau, Germany

**Keywords:** PDAC, Chemotherapy, Outcome, Mortality, Survival, Pancreas surgery

## Abstract

**Background:**

The recommendation for postoperative chemotherapy in pancreatic ductal adenocarcinoma (PDAC) is based on prospective randomized trials. However, patients included in clinical trials do not often reflect the overall patient population treated in clinical practice.

**Materials and methods:**

A retrospective review of all patients undergoing pancreas resection for PDAC between 2001 and 2013 was performed. Follow-up data from oncologists, general practitioners, or hospital patient files were available for 92% of patients.

**Results:**

A total of 251 patients were included in our analysis. Chemotherapy was recommended for 223 patients, but 86 patients did not follow the recommendation. The application of the recommended chemotherapy, consisting of 6 cycles of gemcitabine, was only applied to 45 patients. Forty patients received the recommended number of cycles with dose reduction or prolonged intervals between cycles, and adjuvant chemotherapy was terminated prior to the intended completion of all 6 cycles in 54 patients. Survival of patients after adjuvant chemotherapy was increased compared to that of patients without chemotherapy (with recurrence 25.6 vs. 14.3 months, *p* = 0.001, and without recurrence 27.4 vs. 14.3 months, *p* <  0.001). Terminating chemotherapy prior to completion (*p* = 0.009) as well as a lower number of chemotherapy cycles (*p* = 0.026) was associated with a decreased survival.

**Conclusion:**

Adjuvant chemotherapy improves overall and disease-free survival after curative pancreatic resection, but only a small fraction of patients completes the recommended 6 cycles of adjuvant chemotherapy. Our data indicates that performance status of patients after pancreas resections for PDAC requires not only highly biologically active but also well-tolerated adjuvant chemotherapy regimens.

## Introduction

Pancreatic ductal adenocarcinoma (PDAC) is one of the most aggressive malignant neoplasms with a poor survival rate [1]. The only potential curative treatment is the surgical resection, which can be performed in less than 20% of the patients as the majority of patients are diagnosed at late stages with locally advanced tumors or even with distant metastases [2]. Despite all treatment advances in the field of pancreatic cancer, the 5-year survival is estimated to be as low as 5% [3]. Adjuvant chemotherapy is the standard of care following surgical resection of PDAC, with numerous studies showing improved long-term survival of patients treated with adjuvant chemotherapy [4, 5]. Recommended by guidelines is that, in order to be able to receive adjuvant chemotherapy, patients must have recovered from pancreatic surgery and need to be in a good physical condition [6, 7]. The recovery from surgery is delayed because of postoperative complications that despite reduced mortality remain common. Serious complications are initiated by pancreatic surgery in up to 20% of the patients [7, 8]. As a consequence, 30% of patients who are primarily eligible for adjuvant chemotherapy are never treated, mostly due to the presence of major comorbidities or due to postoperative complications after pancreas resection [7, 9, 10]. But also in patients with complication-free postoperative course, about 40% do not receive the complete treatment or require dose-reduction due to chemotherapy-related toxicity and adverse events [4, 11]. The aim of this single-center study was to evaluate the effect of adjuvant chemotherapy on long-term survival of patients after pancreatic resection for ductal adenocarcinoma. We also focused on the number of chemotherapy applications performed, reasons leading to an early termination of the adjuvant chemotherapy, and impact of the early termination of adjuvant chemotherapy on the survival of the patients.

## Methods

This study is a monocentric, retrospective cohort study at the Department of General and Visceral Surgery of the University Hospital. From November 2001 to December 2012, all patients, undergoing pancreas resection due to pancreatic ductal adenocarcinoma (PDAC) with curative intend, were retrospectively screened. The study was performed according to the principles of the Declaration of Helsinki and was approved by the ethical committee of the Medical Faculty of the University.

### Criteria of inclusion and exclusion

For inclusion, curative intend had to be stated and PDAC had to be histopathologically proven. All subtypes and localizations of ductal adenocarcinoma as well as all kind of surgical procedures were considered. Patients, who received neoadjuvant chemotherapy or radiation, were not enrolled as well as patients, who died during the hospital stay.

### Data acquisition and outcome measures

Data was obtained from in-house medical records and from the database of the comprehensive cancer center, as well as follow-up reports from oncologists and general practitioners. Examined parameters included patients’ demographics; overall, 5-year, and disease-free survival; postoperative complications; and tumor and treatment characteristics such as type and course of operation. Furthermore, the application of adjuvant chemotherapy or reasons for not applying and premature termination of adjuvant chemotherapy were captured. Primary aim was the impact of number of adjuvant chemotherapy cycles on long-term survival of patients after pancreatic resection to clarify the hypothesis whether more cycles might improve survival.

### Statistical analysis

Parameters were documented and analyzed using IBM SPSS for Windows (Version 22.0), and statistical significance was tested using Mann–Whitney *U* test for continuous and the chi-square tests as well as the Fisher’s exact test for categorical variables. Overall survival was analyzed using the Kaplan–Meier method with post hoc log-rank tests. Multivariate survival analysis was performed with the Cox proportional hazard model. *p* <  0.05 was considered significant. Results are presented as median values unless otherwise specified.

## Results

Overall, a total of 251 PDAC patients after pancreatic resection with curative intent were included in the analysis (see Table 1). Follow-up was available for 92% (*n* = 232) of the patients, whereas 80% (*n* = 186) completed 5-year follow-up. Median age was 67 years (range 30–88 years), and slightly more patients were female (*n* = 131, 52%) than male (*n* = 120, 47%). More than three quarters of the patients (*n* = 194, 77%) suffered from cardiovascular, pulmonary, renal, or hepatic diseases leading to an ASA classification of II in 60% and of III in 32% of the cohort [12]. The median duration between diagnosis and operation lasted 23 days (range 3–241 days). TNM classification of patients is shown in Table 2 [13]. Most tumors were located in the head (*n* = 209, 83%), followed by the (*n* = 26, 10%) and body (*n* = 16, 6%). Correspondingly, 190 (76%) patients received pancreatic head resections, 29 (13%) distal pancreas resections and 29 (12%) total pancreatectomies. Tumor diameter was on average 2.7 cm. The median operation duration was 421 min (range 140–717 min). Eighteen (7%) suffered from intraoperative complications and 143 (57%) suffered from diverse postoperative complications, leading to an operative revision in 38 (15%) patients. Patients stayed in the intensive care unit for 5 days (range 1–32 days). The median overall hospital stay was 18 days (range 7–63 days). Drains were removed after 7 days (range 0–62 days).

**Table 1:** Patients’ demographics of all included patients after curative intended pancreas surgery due to pancreatic ductal adenocarcinoma (*n* = 251)

	Median (range)
Age (years)	67 (30–88)
BMI (kg/m^2^)	24.6 (15–39)
	*n* (%)
Gender (female/male)	131/120 (52%/48%)
Smokers	53 (21%)
Alcohol abuse	28 (11%)
ASA Score [12]	
I	13 (5%)
II	152 (61%)
III	81 (33%)
IV	3 (1%)
Common comorbidities	
Hypertension	133 (54%)
Post pancreatitis	125 (50%)
Hepatic disease	100 (40%)
Coronary heart disease	43 (17%)
Pulmonary disease	33 (13%)
Renal insufficiency	30 (12%)
Diabetes	28 (11%)
Localization of tumor	
Head	209 (83%)
Body	16 (7%)
Tail	26 (10%)
Type of surgery	
Pancreatoduodenectomy	190 (76%)
Distal pancreatectomy	29 (13%)
Total pancreatectomy	32 (11%)
Complications	
Intraoperative complication	18 (7%)
Postoperative complication	143 (57%)
Operative revision	38 (15%)
	median (range)
Hospital stay (days)	
Overall	18 (7–63)
Intensive care unit	5 (1–32)
Drain remove (days)	7 (0–62)

**Table 2:** Final TNM classification of pancreatic ductal adenocarcinomas of all included patients after curative intended pancreas surgery (*n* = 251)

		G	T	N	M	Pn	L	V	R
0	*n*			68	242	44	60	163	181
	%			27	96	18	24	65	72
1	*n*	7	6	183	8	157	132	39	67
	%	3	3	73	3	63	53	16	27
2	*n*	158	16					2	3
	%	63	6					1	1
3	*n*	83	218						
	%	33	87						
4	*n*	3	9						
	%	1	4						
	Overall	251	251	251	250	201	192	204	251

*G* grade of tumor cells, *T* tumor size, *N* lymph node manifestation, *M* distant metastases, *Pn* perineural invasion, *l* invasion into lymphatic vessels, *V* invasion into veins, *R* status of resection

### Chemotherapy treatment

**Fig. 1:**
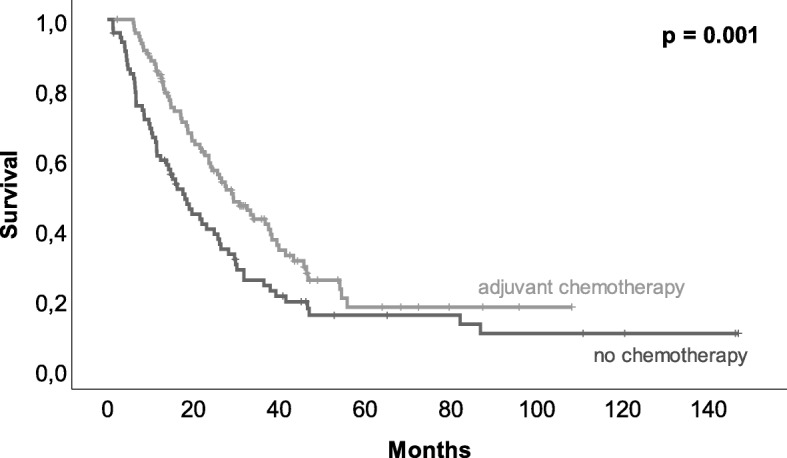
Kaplan–Meier estimator of patients with and without adjuvant chemotherapy. Adjuvant chemotherapy after pancreas surgery due to pancreatic ductal adenocarcinoma led to a significantly better survival (14.3 vs. 25.6 months, *p* = 0.001)

**Fig. 2:**
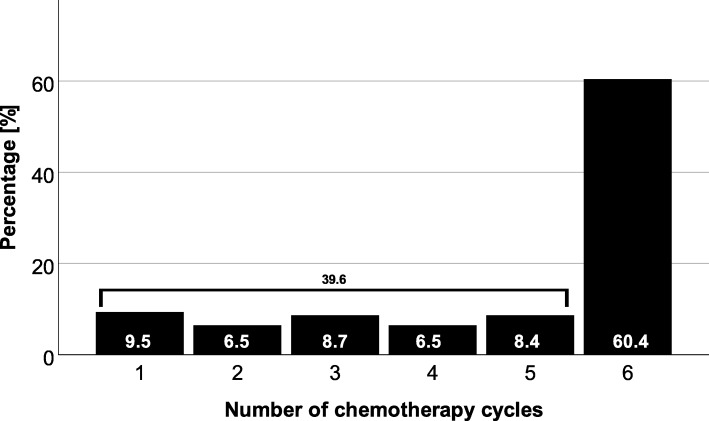
Distribution of patients related to the number of chemotherapy cycles. More than half of patients (60%) received 6 cycles of chemotherapy as it is recommended

**Fig. 3:**
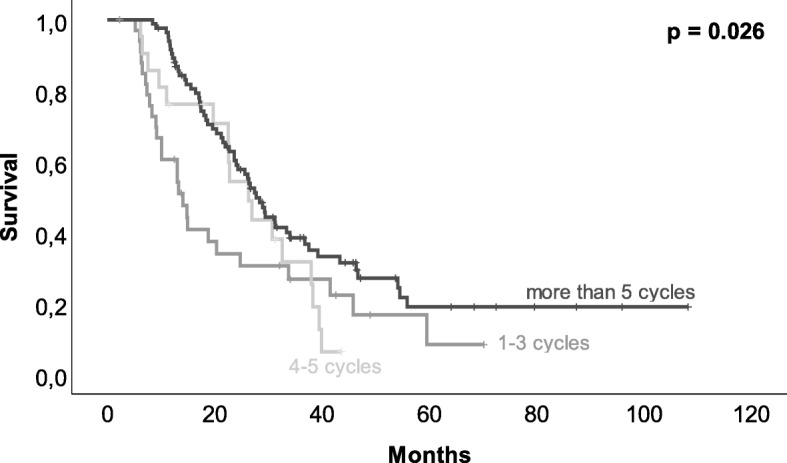
Kaplan–Meier estimator of the relation between the number of chemotherapy cycles and survival. Patients with 6 cycles of chemotherapy showed a median overall survival of more than 27 months, with 4–5 cycles of 26 months and with 1–3 cycles of 14 months (*p* = 0.026)

In 223 (89%) patients, adjuvant chemotherapy was recommended (suggested by interdisciplinary tumor board), but only 62% (*n* = 137) of the patients finally received adjuvant chemotherapy. Two patients (7%) without recommendation for adjuvant chemotherapy received it leaving 139 (56%) patients with adjuvant chemotherapy after pancreas resection. Application of adjuvant chemotherapy had an impact on overall survival (see Fig. 1, *p* = 0.001). In 33% (*n* = 27) of patients with recommended but not delivered adjuvant chemotherapy, tumor recurrence or metastases were detected prior to the initiation of adjuvant chemotherapy. Further reasons for not delivering adjuvant chemotherapy were poor general condition (*n* = 17, 21%) or personal reasons (*n* = 13, 16%). Postoperative complications were only in 9% (*n* = 7) of the patients the cause for not applying adjuvant chemotherapy. On average, chemotherapy was delivered later than recommended. The median for starting adjuvant chemotherapy was 8.5 weeks after the operation while studies recommended 6–8 weeks postoperatively. In contrast to not applying chemotherapy, postoperative complications were frequently the reason for delaying chemotherapy (*p* = 0.011). To that respect, delayed removal of abdominal drains (> 7 days, *p* = 0.011) and prolonged hospital stay (> 18 days, *p* <  0.001) were found responsible. In line with the recommendation in the treatment period, gemcitabine (GEM) was applied as monotherapy in 95% (*n* = 130) of patients. Three (2%) patients had a therapy with the combination of GEM and 5-fluorouracil (5-FU) and two (2%) patients had a monotherapy with 5-FU. Additional two (2%) patients had a therapy with the combination of GEM and erlotinib. More than 60% (*n* = 84) of treated patients received 6 cycles of chemotherapy (Fig. 2). Overall, 54 (39%) out of 139 patients, who received adjuvant chemotherapy, terminated the therapy prior to finishing 6 cycles. Reasons for premature termination of adjuvant chemotherapy were toxicity of the applied medication (*n* = 13, 24%) or recurrence (*n* = 18, 33%). Forty-one percent (*n* = 22) premature termination of chemotherapy was undertaken due to patient wish. When adjuvant chemotherapy was terminated early, recurrence-free survival was decreased from 12.2 to 6.9 months (*p* = 0.005) even when patients with recurrence less than 1 month postoperatively were excluded. In 40 (29%) patients, the anticipated dose of GEM had to be reduced, cycles elongated or, medication had to be changed. Patients with 6 cycles of chemotherapy showed a median overall survival of 27.7 months. Twenty-one (15%) patients that received 4–5 cycles showed a median survival of 26 months, and 33 (24%) of patients receiving only 1–3 cycles had a median survival of 14 months (Fig. 3). Median overall survival was not affected by delayed initiation of chemotherapy (> 8 weeks, *n* = 71, *p* = 0.510) or dose reduction, conversion of medication, or prolonged interval between chemotherapy applications (*n* = 40, *p* = 0.449).

Forty percent (*n* = 56) of patients received adjuvant chemotherapy in our hospital, and 60% (*n* = 83) were treated by an oncologist in private practice. External oncologists performed dose reductions approx. 2.5 times more frequent than oncologists associated to the university hospital. Patients with dose reduction (*n* = 40) were more frequently treated external (*n* = 29, 73%) than in our hospital (*n* = 11, 27%, *p* = 0.097).

### Subgroup analysis of long-term survivors (> 5 years)

Until the end of follow-up, only eight patients (3%) were alive and in complete remission (CR). In all cases, CR lasted for more than 5 years and they did not develop any recurrence. Only four of these patients had full recommended chemotherapy regimen (6 cycles of GEM, full dosage). Patients’ characteristics are shown in Table 3. Due to small sample size, we waived further statistical analysis of these patients.

**Table 3:** Subgroup analysis of long-term survivors in complete remission with (CTx, *n* = 4) and without completion of full recommended chemotherapy regimen (no CTx, *n* = 4)

	CTx	noCTx
	Median (range)	Median (range)
Age (years)	69 (65–70)	57 (30–84)
BMI (kg/m^2^)	23.4 (23–32)	24.5 (23–25)
	*n* (%)	
Gender (female/male)	4/0 (100/0%)	3/1 (75/25%)
Smokers	0 (0%)	1 (25%)
Alcohol abuse	0 (0%)	0 (0%)
ASA Score [12]		
I	1 (25%)	0 (0%)
II	2 (50%)	2 (50%)
III	1 (25%)	2 (50%)
IV	0 (0%)	0 (0%)
Common comorbidities		
Hypertension	1 (25%)	3 (75%)
Post pancreatitis	2 (50%)	2 (50%)
Hepatic disease	1 (25%)	0 (0%)
Coronary heart disease	0 (0%)	0 (0%)
Pulmonary disease	0 (0%)	0 (0%)
Renal insufficiency	0 (0%)	1 (25%)
Diabetes	1 (25%)	0 (0%)
Localization of tumor		
Head	3 (75%)	4 (100%)
Body	1 (25%)	0 (0%)
Tail	0 (0%)	0 (0%)
Grading of tumor		
G2	3 (75%)	4 (100%)
G3	0 (0%)	0 (0%)
G4	1 (25%)	0 (0%)
Invasion of lymph nodes (N)		
N0	2 (50%)	2 (50%)
N1	2 (50%)	2 (50%)
Type of surgery		
Pancreatoduodenectomy	3 (75%)	4 (100%)
Distal pancreatectomy	1 (25%)	0 (0%)
Total pancreatectomy	0 (0%)	0 (0%)
Complications		
Intraoperative complication	0 (0%)	0 (0%)
Postoperative complication	3 (75%)	2 (50%)
Operative revision	0 (0%)	0 (0%)
	Median (range)	Median (range)
Hospital stay (days)		
Overall	17 (14–45)	21 (16–31)
Intensive care unit	5 (3–6)	7 (4–7)
Drain remove (days)	6 (3–45)	7 (5–8)

### Concordance of tumor marker and survival

The pancreas-specific tumor marker CA 19.9 was increased in 165 (66%) patients preoperatively. The median concentration of CA 19.9 was 113 (95% CI 509–1304) U/mL at the time of diagnosis. Preoperative CA 19.9 of more than 500 U/mL (*n* = 61, 27%) was associated with decreased overall survival (12.8 vs. 22.6 months, *p* = 0.012) as well as with decreased 3-year survival rate (8 vs. 27%, *p* = 0.001). The recurrence-free survival of patients with a preoperatively CA 19.9 of more than 500 U/mL was 5.5 (range 5–9) months compared to 10.1 (range 10–14) months of patients with lower values (*p* = 0.475). CA 19.9 decreased to 25 (95% CI 220–1021) U/mL after pancreas resection. Patients (*n* = 80, 38%) without normalization of CA 19.9 (< 37 U/I) postoperatively had a significantly lower overall survival than patients (*n* = 131, 62%) with normalization (*p* = 0.001). Overall survival of patients with normalized CA 19.9 postoperatively was 26.4 (range 7–87) months compared to 17.2 (range 1–82) months of patients without normalization (*p* <  0.001). The recurrence-free survival of postoperatively normalized patients was 12.2 (range 0–82) months compared to 5.3 (range 0–28) months of non-normalized patients (*p* < 0.001). The higher CA 19.9, the shorter was survival (preoperative: *r* = − 0.177, *p* = 0.007, postoperative: *r* = − 0.147, *p* = 0.32). Upon recurrence, CA 19.9 increased to 147 (95% CI 724–3523) U/mL. CA 19.9 of more than 500 U/mL (*n* = 55, 33%) at the time of recurrence diagnosis showed a slightly but non-significant decreased overall survival (13.0 vs. 23.6 months, *p* = 0.081) as well as a significant decreased 3-year survival rate (7 vs. 23%, *p* = 0.01).

### Recurrence and survival

**Fig. 4:**
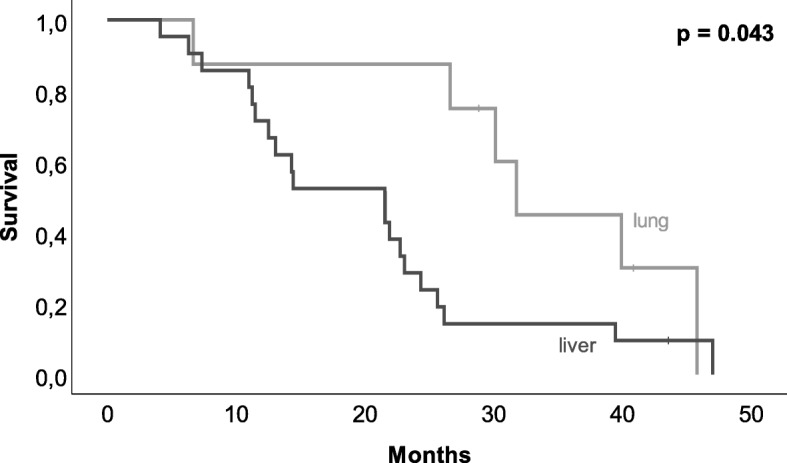
Kaplan-Meier-estimator of site-specific overall surgical, comparison of lung metastasis and liver metastasis. Patients with lung metastases showed a significantly better survival than patients with liver metastasis (*p* = 0.046)

Diagnosis of recurrence was made in 193 (83%) of 232 follow-up data patients with a median disease-free survival of 7.8 months (range 0–83 months) after resection of the pancreatic tumor. Median disease-free survival was significantly affected by adjuvant chemotherapy and found to be 4.1 months after operation in patients without adjuvant chemotherapy (*n* = 84) and 10.9 months in patients with chemotherapy (*n* = 139, *p* = 0.01). The majority of patients (*n* = 85, 44%) suffered from distant metastases, followed by the combination of distant metastases and local recurrence (*n* = 84, 43%). Sole local recurrence was only found in 13% (*n* = 25) of patients. Distant metastases were found in the liver (*n* = 95, 49%), lymph nodes (*n* = 75, 39%), peritoneum (*n* = 71, 37%), and lung (*n* = 50, 26%). Subgroup analysis of influence of site-specific metastases on survival showed that lung metastases were associated with a better survival compared to metastases of the liver (*p* = 0.043, see Fig. 4). Patients with lung metastases (*n* = 11) had an overall survival of 31.0 (range 7–46) months compared to 22.0 (range 4–47) months of patients with liver metastases (*n* = 21).

The median overall survival was 18.5 (range 1–147) months. One-year survival rate was 69%, decreasing to 21% after 3 years and to 6% after 5 years. Almost 90% (*n* = 171, 87%) of the patients died of tumor progression or tumor-related complications. The cause of death of 23 (12%) patients is unknown. Only three patients (1%) died not to tumor-related causes (myocardial infarction, vasovagal asystole, and stroke). The most common tumor-related cause of death was a worsening of general condition presenting as increasing weakness, fatigue, and immobility in 135 patients (69%). Twenty-eight percent (*n* = 55) suffered from tumor cachexia and 12% (*n* = 24) from infections. Nearly half of the deceased (*n* = 92, 46%) had an organ failure such as a respiratory insufficiency (*n* = 36, 18%), renal insufficiency (*n* = 17, 9%), or a liver failure (*n* = 38, 19%). Ascites occurred in 30% (*n* = 58) of the deceased.

### Multivariate risk analysis (see Table 4)

The multivariate analysis showed that the absence of lymph node invasion (*p* = 0.012), of lymph vessel or vein invasion (*p* = 0.014 and *p* = 0.016), and distant metastases (*p* < 0.001) were associated with longer overall survival. A higher tumor grading (G1/G2, *p* = 0.019) as well as R0 resection (*p* < 0.001) improved survival.

**Table 4:** Multivariate risk analysis. Impact of various characteristics on overall survival of all included patients with pancreatic ductal adenocarcinoma after curative intended pancreas surgery (*n* = 251)

		Survival [months]	CI [months]	*p*
Gender	Male	19.6	16.2–23.1	0.705
	Female	21.9	16.8–27.0	
Age [years]	< 63	22.8	14.9–30.7	0.085
	< 72	22.5	17.7–27.4	
	≥ 72	17.6	13.7–21.4	
BMI [kg/m^2^]	> 24,6	18.7	16.1–21.3	0.903
	≥ 24,6	21.9	18.2–25.6	
Weight loss [kg] (preoperatively)	< 5	19.0	14.5–23.5	0.878
	≥ 5	21.2	17.1–25.3	
Comorbidities	Yes	20.7	17.5–24.0	0.464
	No	19.5	7.9–31.2	
Pancreatitis (preoperatively)	Yes	18.5	14.3–22.7	0.395
	No	22.5	18.1–27.0	
Diabetes (postoperatively)	Yes	21.2	15.8–26.5	0.565
	No	20.3	16.2–24.5	
ASA [12]	I	20.4	11.1–29.6	0.732
	II	21.5	17.8–25.2	
	III	19.7	13.5–25.3	
	IV	12.1	3.1–21.1	
Alcohol	Yes	19.7	2.7–23.5	0.585
	No	17.2	17.2–23.5	
Nicotine	Yes	22.8	16.3–29.2	0.661
	No	19.7	16.2–23.2	
Diagnosis to OP [days]	< 23	20.4	16.5–24.2	0.817
	≥ 23	20.4	15.6–25.1	
Duration of OP [min]	< 400	19.6	16.5–22.7	0.505
	≥ 400	21.9	16.8–27.1	
ICU stay [days]	< 5	19.4	13.7–25.1	0.837
	≥ 5	20.4	14.6–26.1	
Hospital stay [days]	< 18	20.8	16.2–25.4	0.147
	≥ 18	20.4	16.4–24.3	
Drain removal [days]	< 7	19.6	15.6–23.5	0.075
	≥ 7	20.4	16.1–24.6	
Complications (intraoperatively)	Yes	17.4	14.2–20.6	0.385
	No	21.2	18.2–24.2	
Complications (postoperatively)	Yes	21.9	15.1–28.7	0.994
	No	20.4	18.2–22.5	
Blood transfusion (intraoperatively)	Yes	13.4	3.0–23.8	0.118
	No	20.8	17.6–24.1	
Re-operation	Yes	15.1	10.1–20.2	0.323
	No	22.5	18.5–24.6	
Adjuvant chemotherapy	Yes	25.6	21.8–29.4	0.001
	No	14.3	11.0–17.7	
Abandonment of chemotherapy	Yes	27.7	22.6–32.8	0.009
	No	19.7	11.3–281	
Cycles	1–3	14	0.2–11.7	0.026
	4–5	26.3	3–20.4	
	6	27.7	1.7–24.4	
Rehabilitation	Yes	21.5	18.3–24.8	0.159
	No	15.1	8.2–22.1	
OP to rehabilitation [days]	< 21	23.6	14.9–32.3	0.419
	≥ 21	18.5	16.5–20.5	
Tumor localization	Caput	20.4	17.2–23.5	0.515
	Corpus	19.4	6.2–40.6	
	Caudae	22.6	11.3–33.8	
Tumor size	T1	38.3	19.9–56.6	0.212
	T2	20.7	15.7–25.8	
	T3	19.7	16.1–23.3	
	T4	10.1	0–21.9	
Tumor grade	G1/G2	21.6	16.7–26.4	0.019
	G3/G4	15.2	7.3–23.1	
Lymph node invasion	N0	30.6	24.2–37.6	< 0.001
	N1	17.3	1.8–13.8	
Lymph vessel invasion	L0	26.6	12.3–41.0	0.014
	L1	17.4	13.3–21.6	
Vein invasion	V0	22.6	18.5–26.6	0.016
	V1	14.4	11.5–17.4	
	V2	10.3	n/c	
Perineural invasion	Pn0	21.6	15.3–27.8	0.242
	Pn1	19.6	14.9–24.3	
Distant metastases	M0	20.8	17.7–24.0	0.012
	M1	6.6	1.4–17.4	
Resection	R0	24.3	19.6–29.2	< 0.001
	R1	13.3	10.7–15.9	
	R2	14.4	17.3–23.4	
CA 19.9 [U/mL] (time of diagnosis)	< 37	23.6	21.3–33.4	0.071
	< 100	27.6	24.3–41.0	
	< 500	18.0	18.0–26.3	
	< 1000	14.1	13.3–23.5	
	≥ 1000	10.4	9.3–27.5	
CA 19.9 [U/mL] (postoperatively)	< 37	26.4	25.3–32.8	0.031
	< 100	15.9	12.2–28.4	
	< 500	14.4	12.3–22.5	
	< 1000	13.0	− 11.3 to 44.2	
	≥ 1000	9.9	7.2–18.3	
CA 19.9 [U/mL] (time of recurrence)	< 37	25.2	21.8–35.4	0.193
	< 100	22.5	19.0–29.3	
	< 500	23.7	20.8–31.1	
	< 1000	16.3	14.9–25.0	
	≥ 1000	10.3	10.1–18.0	

Reference range of CA 19.9 < 37 U/mL

*BMI* body mass index, *CI* confidence interval, *n/c* not calculated, *OP* operation

### Discussion

Prognosis of PDAC is, due to late diagnosis, rapid tumor progression, and frequent recurrence, limited. Pancreatic surgery followed by adjuvant chemotherapy improves survival and is the only chance for curative treatment. Despite a defined and guideline-recommended therapeutic strategy, the prognosis remains poor. It is known that postoperative complications and a delayed recovery after pancreatic surgery lead to adjuvant chemotherapy omission and treatment delays [7]. Initiation of adjuvant chemotherapy is recommended 6–8 weeks postoperatively, which was, due to various factors, frequently not able in our patients. Delayed initiation of chemotherapy has been reported previously. Merkow et al. observed an initiation of adjuvant chemotherapy with a median time to adjuvant therapy of 52 days (7.4 weeks) in patients without postoperative complications. In patients with complications, the delay was even 70 days [7]. The retrospective analysis of clinical data reveals that the recommended duration between surgery and initiation of chemotherapy is likely to be met only in a subset of patients. Nevertheless, survival analysis of our data showed no association between delayed treatment and decreasing survival, which is confirmed by other studies [14]. Timing of chemotherapy is not as crucial as an adequate number of cycles [14, 15]. Our results showed that more than 3 cycles after curative pancreas surgery were associated with better survival. Interestingly, the benefit of more than 5 cycles was smaller and patients with 4–5 and with 6 cycles had a comparable survival. Contrary to that, Epelboym et al. found that survival is increased after 6 cycles or more cycles of adjuvant chemotherapy [15]. However, they only differentiated between 1 and 5 and 6 and more cycles, which is different to our analysis. For other cancers, it has been reported that more cycles do not necessarily lead to an increased survival [16, 17]. Nevertheless, it must be considered that the results might be influenced by patients’ comorbidities. Only patients in good condition are able to be treated with chemotherapy. It is even more surprising that patients treated with 4–5 cycles had nearly the same survival time than patients with 6 cycles as it is assumable that the patients with fewer cycles quitted therapy due to poor condition or intolerable side effects.

Subgroup analysis of lung metastatic patients compared to liver metastatic patients showed site-specific influence on survival. Despite small sample size, we found an improved overall survival of patients with lung metastases compared to patients with liver metastases, confirming the results of a previously published database analysis of metastatic pancreatic cancer patients with real-life data of initially curative treated patients [18]. Further analysis of our patients showed just tendencies of demographic and clinical differences prior to diagnosis of pancreatic cancer, but due to the small sample size, we are not able to draw firm conclusions. However, future research should include molecular genetic data [19], which is not available in the patients of our study, who were treated between 2001 and 2012, where molecular genetic analysis was not performed as standard.

Due to the retrospective nature, GEM had been applied to 95% of the patients which was in line with past guidelines for chemotherapy treatment of PDAC [4, 20]. Based on novel findings and significantly improved survival, this recommendation will change to a modified treatment with FOLFIRINOX [21, 22]. Our retrospective analysis, however, indicated that already a substantial number of patients are not able to receive full guideline-recommended chemotherapy. For these patients, the beneficial toxicity profile of GEM will remain a valid option for adjuvant chemotherapy. Additionally, our data questions the general applicability of adjuvant mFOLFIRINOX in the overall cohort of resected pancreatic cancer patients since 62% of our patients received adjuvant chemotherapy at all and only 20% received all cycles without dose reduction. Our data is supported by another publication reporting adjuvant chemotherapy in slightly more than half of the patients after curative intended pancreatic surgery [7].

### Limitations

Our retrospective analysis is limited by its monocentric study design and a small sample size as well as by lack of documentation and documentation errors, but only 8% of patients were lost to follow-up. The large time span for collecting the data and change of guidelines affected the treatment recommendations given and the intensity by which the indication for adjuvant therapy was explained to the patient. For identifying reasons for terminating chemotherapy, heterogeneous documentation received from different sources had to be used.

## Conclusion

Adjuvant chemotherapy improves long-term and disease-free survival after curative pancreatic resection, but only a small fraction of patients completes the recommended 6 months of adjuvant chemotherapy. Our data indicates that well-tolerated adjuvant chemotherapy with gemcitabine will remain a valuable tool of adjuvant therapy in pancreatic cancer in patients with low-performance status also in times of more effective chemotherapy regimens.

## Data Availability

The datasets used and analyzed during the current study are available from the corresponding author on reasonable request.

## Abbreviations

5-FU: 5-Fluorouracil
GEM: Gemcitabine
PDAC: Pancreatic ductal adenocarcinoma
